# Caesium and iodine release from spent mixed oxide fuels under repository relevant conditions: Initial leaching results

**DOI:** 10.1557/s43580-022-00220-7

**Published:** 2022-02-09

**Authors:** Christian Schreinemachers, Gregory Leinders, Thierry Mennecart, Christelle Cachoir, Karel Lemmens, Marc Verwerft, Felix Brandt, Guido Deissmann, Giuseppe Modolo, Dirk Bosbach

**Affiliations:** 1 Forschungszentrum Jülich GmbH, Institute of Energy and Climate Research, IEK-6: Nuclear Waste Management and Reactor Safety, 52425 Jülich, Germany; 2 Belgian Nuclear Research Centre (SCK CEN), Institute for Nuclear Materials Science, Boeretang 200, 2400 Mol, Belgium; 3 Belgian Nuclear Research Centre (SCK CEN), Institute for Environment, Health and Safety, Boeretang 200, 2400 Mol, Belgium

## Abstract

Autoclave leaching experiments are conducted on three well-characterised, irradiated, and cladded mixed oxide fuel-rod segments with burnups ranging from 29 GWd/t_HM_ to 52 GWd/t_HM_ to investigate the instant release fraction of fission gases and long-lived fission products and to assess the long-term fuel matrix corrosion. The segments are exposed to bicarbonate solutions as reference groundwater at neutral pH and a synthetic young cementitious water at pH 13.5 under reducing atmosphere (4 vol% H_2_ in Ar at 40 bar pressure), since 2018. The initial leaching results for the fission products caesium and iodine as representative elements of the instant release fraction were found to depend on the leachate composition as well as on the fuel burnup.

**Graphical abstract:**

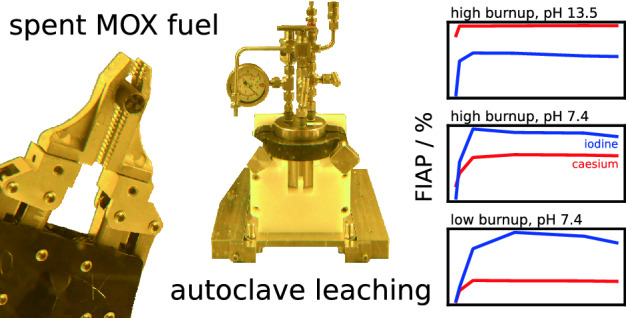

## Introduction

Disposal in a deep geological repository (DGR) based on a multi-barrier concept is considered as the safest and most sustainable option for the management of spent nuclear fuels (SNFs) in many countries. Demonstrating the long-term safety of a DGR for SNFs over assessment time-frames of up to one million years requires a profound understanding of the corrosion behaviour of SNF coming into contact with groundwater, when the waste canister is eventually breached. During the last decades, multiple studies addressed this topic, leading to a good phenomenological understanding of the long-term behaviour of SNF in a DGR [1–5]. However, various processes contributing to the (radiolytic) matrix corrosion of SNF in the generally reducing repository environment are still not fully understood, and corrosion data on spent mixed oxide (MOX) fuels are scarce to date.

The SF-ALE project (spent fuel autoclave leaching experiments) was initiated between the Belgian Nuclear Research Centre (SCK CEN) and Forschungszentrum Jülich GmbH to investigate effects of environmental conditions on the corrosion of various types of SNF. Within SF-ALE, the release of radionuclides from irradiated MOX fuel-rod segments exposed to a bicarbonate solution (BIC) as reference groundwater and a synthetic young cementitious water (YCW) under reducing atmosphere is investigated. The experimental phase started in 2017 and consists of three work packages: (1) MOX post-irradiation examination, (2) autoclave leaching study, and (3) post-leaching characterisation. In this work, we present initial results of the release of the fission products caesium and iodine from the MOX during the first two years of leaching.

## Materials and methods

### MOX fuel-rod properties and sample segments

**Fig. 1:**
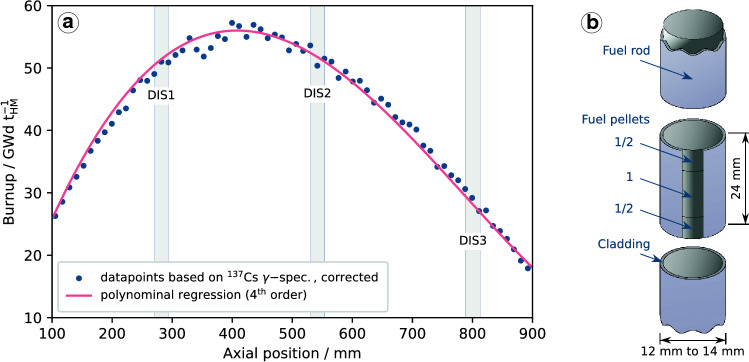
Burnup of the irradiated MOX fuel-rod as function of the axial position, as well as regions of segments in use within the leaching phases of SF-ALE (**a**). Schematic representation of a fuel-rod segment studied within the autoclave leaching experiments (**b**)

A MOX fuel-rod with *Zircaloy-4* cladding is studied within SF-ALE. The fuel pellets were fabricated by Belgonucleaire (Dessel, Belgium) via the MIMAS process [6]. Initially, it contained a Pu molar metal fraction of 14 mol% and an oxygen to metal ratio of 1.99 [7]. The fuel-rod was irradiated in the Belgian reactors BR-3 and BR-2 for a total duration of 1048 days between 1986 and 2011, employing an average linear power rating of about 210 W/cm with a maximum of about 325 W/cm. Its burnup was estimated non-destructively by γ-spectrometry, taking a fission energy of 208 MeV/fission and an axial depending $^{137}$Cs in-pile decay factor into account [8]; the result is shown in Fig. 1a.

Three fuel-rod segments with burnups of about 50 GWd/t$_{\text {HM}}$, 52 GWd/t$_{\text {HM}}$, and 29 GWd/t$_{\text {HM}}$ were selected for the autoclave leaching experiments. The segments are indicated in Fig. 1a and labelled with *DIS1*, *DIS2* and *DIS3*, respectively. During the segment preparation, the fuel-rod was cut in such a way that they halved a pellet. The segments used in the leaching experiments have lengths of 24 mm and contain two half pellets and one entire pellet in between, as schematically shown in Fig. 1b.

### Autoclave leaching experiments

The cladded fuel segments are exposed to either YCW or BIC, the latter has an initial pH of 7.4 and contains 1 mmol/L NaHCO_3_ and 19 mmol/L NaCl. The YCW has a pH of 13.5 and contains K, Na, and Ca, as well as traces of Si, Al and B, with contents comparable to those reported by Ref. [9]. In order to investigate the influence of the leachate on the dissolution behaviour, the high burnup segments (*DIS1* and *DIS2*) are leached in YCW and BIC, respectively. The segment with the lower burnup (*DIS3*) is exposed to BIC to study the influence of the fuels’ burnup.

The experimental set-up is based on a design developed at the *Karlsruhe Institute of Technology* [10] and the experiments are carried out at room temperature in autoclaves manufactured by Berghof ($V_{\text {total}}$ = 250 mL) [7]. The latter contain a titanium liner, which is filled with the fuel-rod segment (12.9 g to 15.7 g) mounted in a holder, the leachate (200 mL), and an Ar:H_2_ gas atmosphere (96:4, $p=40\,\text {bar}$).

The autoclave experiments were initiated in September 2018 and two leaching phases with durations of 18 months were envisaged. Samplings of gas (50 mL) and leachate (10 mL) are carried out within the course of each phase. The first sampling was performed after 5 days and the solution was replaced with fresh leachate. Further samplings were done after 3 weeks, 3 months, and 9 months. A sampling and replacement of the autoclaves’ titanium insert and the leachate was foreseen after 18 months as end of the first phase. The sampling was shifted to month 20 and the pressure was reduced to 4 bar as safety precaution until the inserts could be replaced (SARS-CoV-2 interruption), which was done after 24 months. The leachate was sampled prior to the exchange of the inserts. Afterwards, fresh leachate was added and the Ar:H_2_ pressure was set to 40 bar. The used inserts underwent an acidic rinsing and the rinsing solution was analysed as well. The transition from the first to the second leaching phase is described in more detail elsewhere [8]. Within phase two, samplings were performed after total durations of 25 months, 30 months, and 36 months. A last sampling is foreseen after 42 months and will complete the second leaching phase in March 2022.

#### Characterisation of leaching samples

Gas samples are analysed to study the release of the fission gases Kr and Xe, combined with the additional monitoring of H_2_, N_2_ and O_2_ [8]. The aqueous samples are analysed for about 30 relevant actinides, fission and activation products by α- and γ-spectrometry, liquid scintillation counting, as well as inductive coupled plasma mass spectrometry (ICP-MS).

In this work, leaching results of the fission products caesium and iodine are presented. The molar caesium concentrations are based on ICP-MS analyses of $^{133}$Cs and $\gamma$-spectrometry results of $^{134}$Cs and $^{137}$Cs, while the molar iodine concentrations originate from ICP-MS analyses of $^{129}$I. The data of ^137^Cs and ^129^I were converted into molar amounts and normalised to the initial molar amounts of the corresponding nuclides in the fuel-rod segment in order to determine their inventory in the aqueous phase (FIAP). The initial amounts of the nuclides originate from depletion calculations [8].

## Results

The burnup results (Fig. 1) show an axial dependency caused by different flux profiles of the reactors BR-2 and BR-3. Consequently, the nuclide inventory required to calculate the FIAPs depends on the axial position as well. The initial caesium content in the segments *DIS1*, *DIS2* and *DI3* was 2.75 mg/g${_\text {fuel}}$, 2.82 mg/g${_\text {fuel}}$ and 1.58 mg/g${_\text {fuel}}$, respectively; the $^{129}$I content was determined to be 0.31 mg/g${_\text {fuel}}$, 0.32 mg/g${_\text {fuel}}$, and 0.18 mg/g${_\text {fuel}}$ [8].

**Fig. 2:**
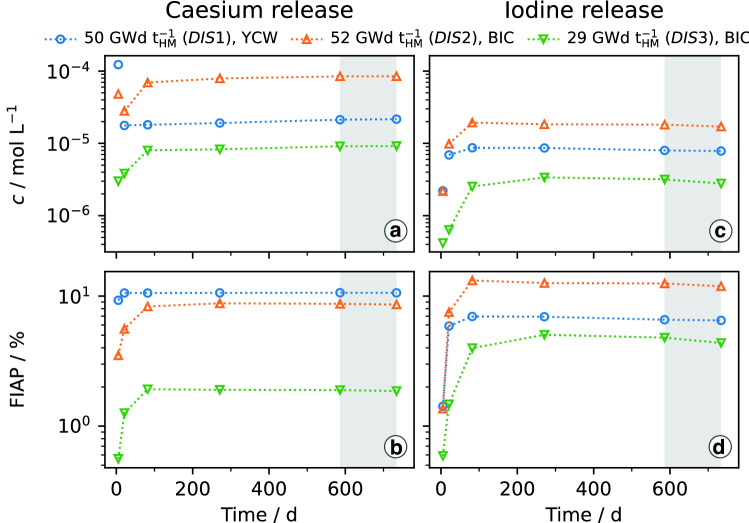
Molar caesium and iodine concentrations obtained in autoclave leaching experiments on spent MOX fuels under reducing conditions (4 vol% H_2_ in Ar at 40 bar pressure; YCW: young cementitious water, BIC: bicarbonate solution) as function of the exposure time (**a**, **c**) and resulting FIAPs for both elements (**b**, **d**). The shaded areas correspond to the 4 bar interval at the end of leaching phase one. Please note that in the top figures (**a**, **c**) the leachate renewal took place after the first data-point, which is disconnected from the subsequent data-points. The bottom row figures (**b**, **d**) represent the integral release (i.e. the release of the first 5 days is added to the subsequent release)

The leaching results for caesium and iodine obtained in the first leaching phase of SF-ALE are summarised in Fig. 2. FIAPs presented in Fig. 2 are semi-cumulative: The data-point determined in the replaced leachate after 5 days was added as offset to the data-points of the renewed leachate.

### Caesium release

After an exposure time of 5 days, the highest caesium content was found in YCW (Fig. 2a, b). The concentrations determined in the renewed YCW were relatively constant from day 21 until the end of leaching phase one and correspond to an average caesium FIAP of 10.6 % for *DIS1*. In the experiments in BIC, a proportional behaviour was observed and a steady state was reached after 82 days of exposure with average caesium FIAPs of 8.6 % and 1.9 % for *DIS2* and *DIS3*, respectively.

Sorbed Caesium masses were determined based on the analysis results of the rinsing solutions. The results were converted into the corresponding caesium FIAPs and taken into account in the last data-points presented in Fig. 2b, but with contributions ranging from 0.01 % to 0.03 % they can be considered as negligible.

### Iodine release

Similar iodine concentrations were obtained in the YCW and BIC leaching samples of the high burnup segments after 5 days (Fig. 2c, d). In the renewed leachates of both experiments, the concentrations increased and reached maxima after an exposure of 82 days, corresponding to FIAPs of 7.0 % and 13.1 % for *DIS1* and *DIS2*, respectively. The leaching of segment *DIS3* took 271 days until a maximum with a FIAP of 5.0 % was observed. A decrease within the uncertainty ranges was noted after surpassing the maxima, which was more pronounced in BIC. However, it occurred for all three experiments and FIAPs of 6.5 %, 11.9 % and 4.4 % were determined after 734 days for *DIS1*, *DIS2* and *DIS3*, respectively.

Due to the instability of iodine in the acidic rinsing solution, it was not analysed in the rinsing samples. Consequently, the FIAPs presented in Fig. 2d do not consider any sorption of iodine to the autoclave liners, which in turn would also be not expected.

## Discussion

In all experiments, a fast initial release of caesium and iodine was observed, reaching nearly constant concentration levels after 82 days (Fig. 2). An influence of the leachate composition on the leaching behaviour of caesium and iodine from irradiated MOX under reducing atmosphere was revealed by several observations: The instant release of caesium occurred within 21 days in YCW, which was delayed to 82 days and by a factor of about 0.8 less pronounced in BIC for comparable burnups of about 50 GWd/t$_{\text {HM}}$. The release of iodine behaved inversely, by a factor of about 1.9 larger fractions iodine were monitored in BIC as compared to YCW, but the dissolution rates were less affected by the leachate variation than the caesium dissolution rates for segments with similar burnups.

A comparison of the leaching results of the segments with varying burnups in BIC shows an influence on the iodine leaching behaviour by a prolonged release within 271 days for the lower burnup of 29 GWd/t$_{\text {HM}}$. Moreover, the iodine FIAP was about a factor of 2.6 higher in the leachate of the segment with a burnup of 52 GWd/t$_{\text {HM}}$, which was in the case of caesium a factor of about 4.6 higher and hence more pronounced.

## Conclusions

Autoclave leaching results showed an influence of the leaching medium and the fuels’ burnup on its leaching behaviour for the release of the fission products caesium and iodine from irradiated, cladded MOX fuel-rod segments under reducing atmosphere. Caesium and iodine release occurred for a fuel burnup of about 50 GWd/t$_{\text {HM}}$ rapidly in less than 82 days in YCW and BIC. For a lower burnup of 29 GWd/t$_{\text {HM}}$ the caesium release rate into BIC was comparable, while iodine release occurred in less than 271 days from the identical segment in the same medium. Furthermore, no significant caesium sorption was noted, demonstrating that caesium remains in aqueous phase for solutions with initial pH values of 7.4 and 13.5 during a time-frame of about two years.

## Data Availability

The data of the current study are available from the corresponding author on reasonable request.
